# Case Report: Complete Maternal Uniparental Disomy of Chromosome 2 With a Novel *UNC80* Splicing Variant c.5609-4G> A in a Chinese Patient With Infantile Hypotonia With Psychomotor Retardation and Characteristic Facies 2

**DOI:** 10.3389/fgene.2021.747422

**Published:** 2021-09-14

**Authors:** Yilun Tao, Dong Han, Yiju Wei, Lihong Wang, Wenxia Song, Xiaoze Li

**Affiliations:** ^1^ Medical Genetic Center, Changzhi Maternal and Child Health Care Hospital, Changzhi, China; ^2^ Department of Pediatrics, Penn State Health Hershey Medical Center, Penn State College of Medicine, Hershey, PA, United States; ^3^ Department of Pediatrics, Changzhi Maternal and Child Health Care Hospital, Changzhi, China; ^4^ Obstetrics Department, Changzhi Maternal and Child Health Care Hospital, Changzhi, China

**Keywords:** maternal uniparental disomy, chromosome 2, UNC80 gene, SNP array, whole exome sequencing

## Abstract

**Background:** Infantile hypotonia with psychomotor retardation and characteristic facies 2 (IHPRF2) is a rare autosomal recessive neurodevelopmental disorder caused by mutations in the *UNC80* gene. It is characterized by severe global developmental delay, poor or absent speech and absent or limited walking abilities. The current study explored a case of a Chinese patient with IHPRF2 caused by a novel splicing variant of *UNC80*.

**Case Report:** The proband is a 8-year-old Chinese male manifested with global developmental delay, severe truncal hypotonia, absent speech and intellectual disability. SNP array analysis revealed a uniparental isodisomy of the entire chromosome 2 [UPD(2)] in the proband. Whole exome sequencing (WES) subsequently identified a novel mutation c.5609-4G>A in the *UNC80* gene*,* which was inherited from his mother and was confirmed by Sanger sequencing, indicating that UPD(2) was of maternal origin.

**Conclusion:** A novel *UNC80* homozygous splicing variant c.5609-4G>A associated with maternal UPD(2) was identified. These findings indicate that UPD poses a high risk of autosomal recessive diseases, and provides information on the variant spectrum for *UNC80*. Our findings elucidate on understanding of the genotype-phenotype associations that occur in IHPRF2 patients.

## Introduction

Infantile hypotonia with psychomotor retardation and characteristic facies 2 (IHPRF2, MIM 616801) is a rare autosomal recessive neurodevelopmental disorder with onset at birth or in early infancy (Shamseldin et al., 2016). It is a phenotypically heterogeneous disease characterized by severe global developmental delay, hypotonia, facial dysmorphism, intellectual disability, poor or absent speech and lack of or limited walking abilities (Perez et al., 2016; Shamseldin et al., 2016; He et al., 2018). The first case of IHPRF2 was reported in Bedouin families in 2016 (Perez et al., 2016). It has previously been reported that IHPRF2 is caused by homozygous or compound heterozygous mutation in *UNC80* gene (MIM 612636) (Lu et al., 2010).

The *UNC80* gene is located on chromosome 2 and comprises of 64 exons. *UNC80* gene encodes a subunit of the non-selective sodium leak channel (NALCN) complex, consisting of 3,258 amino acids (Lu et al., 2010). This channel complex is a voltage-insensitive and nonselective sodium-leak channel, which is predominantly expressed in the brain and plays an important role in the establishment and maintenance of resting membrane potentials in neuron (Lu et al., 2007; Zhang et al., 2021). Proteins expressed from the *UNC80* gene interacts with NALCN and acts as a scaffold protein for UNC79 (Lu et al., 2010; Wie et al., 2020). Recessive loss-of-function biallelic pathogenic variants of *UNC80* cause UNC80 deficiency, which can result in dysfunctional NALCN complex leading to severe pathological phenotypes (Valkanas et al., 2016; Wie et al., 2020).

Uniparental disomy (UPD), a rare case that was first reported in 1980, occurs when a chromosome pair is derived from the same parent in a disomic cell with a balanced karyotype (Engel 1980). The prevalence of UPD ranges from 1 in 2000 to 1 in 5,000 people (Robinson, 2000; Liehr, 2010; Nakka et al., 2019). UPDs can cause clinical abnormalities, resulting in an aberrant dosage of imprinting genes or homozygosity of variants for recessive phenotypes (Yamazawa et al., 2010). Maternal and paternal UPDs have been reported in almost every human chromosome (Nakka et al., 2019). However, maternal UPD of chromosome 2 [UPD(2)mat] with a homozygous pathogenic variant in *UNC80* has not been previously reported.

We report a case of IHPRF2 with a novel homozygous splicing variant c.5609-4G>A of *UNC80* gene arising from UPD(2)mat, which expands on the disease spectrum associated with *UNC80* mutations. In addition, previous genotypes and phenotypes of patients with IHPRF2 were reviewed, to help in understanding the genotype-phenotype relationship of *UNC80*.

## Materials and Methods

### Case Report

The proband is an 8-year-old male born as the sole child of healthy and non-consanguineous Chinese parents. The mother did not report any history of exposure to teratogenic pathogens or drugs during gestation. Birth weight of the child was 3,150 g (25th percentile), birth length was 51 cm (75th percentile), and occipitofrontal circumference (OFC) was 36 cm (>95th percentile) on week 39 of gestation. He has been suffering from severe hypotonia and feeding difficulty since birth. The proband was able to grasp at 6 months and could sit at 9 months. At 13 months of age, the patient was admitted to Beijing Children’s Hospital affiliated to Capital Medical University for astasia, development delay and failure to thrive with feeding difficulty. He manifested dystonia in the extremities, with complete inability to stand up and walk. He could not speak, even simple words such as “baba” and “mama”. Electroencephalograph (EEG) analysis did not reveal any abnormalities, whereas brain magnetic resonance imaging (MRI) showed bilateral dilation of lateral ventricles, periventricular leukomalacia and delayed myelination. No definite diagnosis existed to that time, and he did not undergo any adjuvant therapy. At the age of five, he presented to our hospital for severe psychomotor development. On examination, he had severe intellectual disability, hypotonia, strabismus and esotropia. He was unable to speak or communicate. He was able to walk with some aid but exhibited poor balance and he could not jump on one foot. In addition, the subject suffered from constipation. Facial dysmorphisms included a triangular face, microcephaly, low-set posterior rotated ears, and a thin and tented upper lip. He had long and slender fingers. His height, weight and head circumference were 107 cm (14th percentile), 15.5 kg (4th percentile) and 47.1 cm (<1st percentile), respectively.

In this family, we identified only one patient (proband) and proband’s father and mother is normal. We therefore used SNP array analysis and whole genome sequencing to search for any evidence of the disease.

### SNP Array Analysis

A DNA sample (250 ng) was extracted from the peripheral blood of the patient and was hybridized using an Affymetrix CytoScan^®^ 750K array kit (Affymetrix, Inc., Santa Clara, CA, United States) according to the manufacturer’s protocol. The SNP array data was analyzed for the presence of copy number variations (CNVs) using Affymetrix Chromosome Analysis Suite (ChAS) Software version 3.3. Pathogenicity of CNVs was evaluated based on published literature and public databases, including DGV (http://dgv.tcag.ca/dgv/app/home), Clingen (https://www.clinicalgenome.org/), DECIPHER (https://decipher.sanger.ac.uk/) and OMIM (https://www.omim.org/). This analysis was performed in accordance with the American College of Medical Genetics and Genomics (ACMG) and the Clinical Genome Resource (ClinGen) 2020 guidelines (Riggs et al., 2020).

### Whole-Exome Sequencing

Peripheral blood sample of the patient was collected. Genomic DNA was extracted using a QIAamp DNA Mini Kit (Qiagen, China), according to the manufacturer’s instructions. DNA library preparation was performed following Illumina protocols, which included end repair, adapter ligation and PCR enrichment. The amplified DNA was then captured using Whole Exome Capture Kit (MyGenostics Inc, Beijing, China). Biotinylated capture probes were designed to tile all exons without repeated regions. The enriched libraries were sequenced for paired-end reads of 150 bp using the Illumina HiSeq X Ten platform.

The bioinformatics analyses were carried out utilizing established methods with some modifications (Zheng et al., 2018; Dai et al., 2019; Han et al., 2020; Zhang et al., 2020). After sequencing, clean reads were aligned to the UCSC hg19 human reference genome using the Burrows-Wheeler Alignment tool. Duplicated reads were removed using the Picard tool (http://broadinstitute.github.io/picard). The variants of SNP and small insertions or deletions (InDel) were detected by GATK HaplotypeCaller, then using GATK VariantFiltration to filter variant. The identified variants were then annotated using ANNOVAR. The variants with frequencies less than 0.05 in the normal population database were screened out, including the 1,000 Genomes, Exome Aggregation Consortium (ExAC), GnomAD and Inhouse database (MyGenostics Inc, Beijing, China). In addition, the identified variants were predicted using Mutation Taster (MT), Sorting Intolerant From Tolerant (SIFT), PolyPhen-2 (PP2) and Genomic Evolutionary Rate Profiling (GERP++), dbscSNV, SPIDEX, SPliceAI. Classification of variants (pathogenic, likely pathogenic, VUS and likely benign and benign) has been done according to the variant interpretation guidelines of American College of Medical Genetics and Genomics (Richards et al., 2015). Finally, we compared the variants found in patient and his parents. The function of the variant and their correlation with the disease phenotype was done by previously published articles and OMIM database.

### Sanger Sequencing

Candidate variable sites were confirmed by Sanger sequencing for the patient and his parents. The primers are as follows: F 5′- CAA​CGA​AGA​GAA​CAA​ACA​CCT​ACG -3′, R 5′- TAT​TGG​AGG​GCA​TTG​AGT​TGC-3. The reference sequence NM_032,504 of *UNC80* was used. Target sequences were sequenced on an ABI 3730 genetic analyzer (Applied Biosystems, Foster City Carlsbad, CA, United States) and identified using Chromas 2.6.5 (Technelysium Pty Ltd, Australia).

## Results

**FIGURE 1:**
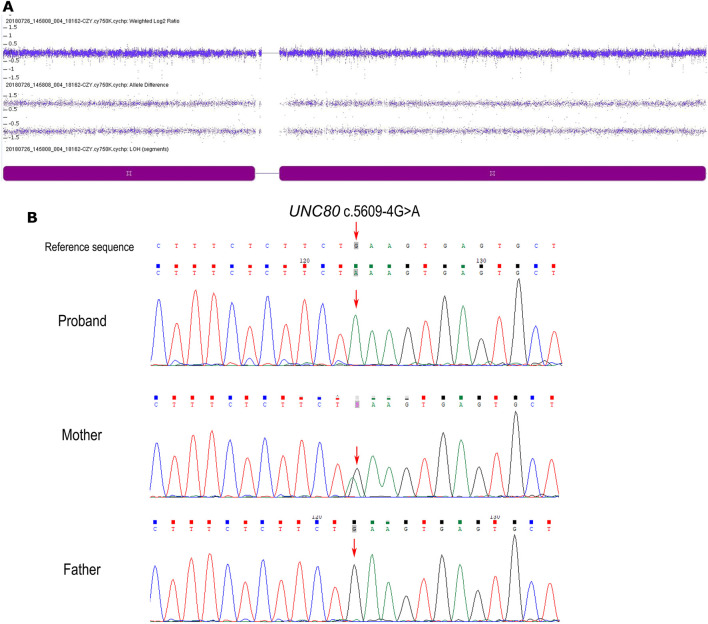
**(A)** Single nucleotide polymorphism microarray (SNP array, Affymetrix Cytoscan 750k) profiles of chromosome 2 in the patient. The dark purple block indicates complete UPD in chromosome 2, with two allele difference tracks where the plot has a line at + 1 and −1. **(B)** Sequence electropherogram of the c.5609-4G> A variation showing (arrow) the homozygous mutation in the patient, the heterozygous genotype in his mother, and wild-type in his father.

Genome-wide SNP array analysis did not reveal any clinically significant copy number variations. However, it revealed an absence of heterozygosity (AOH) across the entire chromosome 2 (Figure 1A). There was no disease-related imprinting gene located on chromosome 2, therefore, whole exome sequencing (WES) of the patient was subsequently performed. A novel homozygous variant c.5609-4G> A in *UNC80* was detected in the proband, which was only present in his mother as a heterozygous trait and was absent in the father (Figure 1B). This contradiction to Mendelian inheritance indicates that the AOH originated from maternal UPD. The variant c.5609-4G> A was located on intron 35, -4 bp to exon 36 splice acceptor site of the *UNC80* gene. This variant has not been previously reported and was absent on the 1,000 Genome Project, the ExAC, the gnomAD database or our inhouse database. Furthermore, it has not been reported in HGMD. *In silico* analysis by MutationTaster and regSNP-intron showed that the variant may have an impact on pre-mRNA splicing (the score was 0.96 and 0.37), whereas SPliceAI indicated a prediction that is in total disagreement. Samples were not available for reanalysis, therefore, RT-PCR was not performed to explore the effect of splicing. Based on ACMG guidelines, the c.5609-4G>A variant was classified as variant of uncertain clinical significance (PM2). The diagnosis of IHPRF2 was confirmed by molecular and clinical findings.

## Discussion

The current study reports a case of an IHPRF2 patient with global developmental delay, truncal hypotonia, intellectual disability, and absent speech. A novel *UNC80* homozygous splicing variant c.5609-4G> A originating from UPD(2)mat was identified, which has not been previously reported, and was absent in the general population. Bioinformatics analysis showed that the variant might be involved in splicing modulation. Similar variants, which lead to protein truncation have been previously reported (Anna and Monika, 2018; Li et al., 2019; Śmigiel et al., 2020). And *in vitro* cytology experiments have demonstrated that loss-of-function mutations in *UNC80* are associated with IHPRF2 (Valkanas et al., 2016; Wie et al., 2020). Clinical presentation of the patient showed that he had IHPRF2. These findings indicate that the variant c.5609-4G> A is associated with pathogenesis of IHPRF2. However, further functional studies are needed to validate the pathogenicity of this variant.

The potential harmful effects of UPD include imprinted gene diseases, or activation of recessive pathogenic genes. Maternal and paternal UPD2, with normal phenotypes, have been reported previously, indicating the absence of genomic imprinting effects in UPD2 (Keller et al., 2009; Ou et al., 2013; Zhou et al., 2017; Zhang et al., 2019). Single conventional SNP-array analysis did not provide a conclusive diagnosis for the patient, therefore, an integrated approach is needed to determine the underlying genetic cause. This limitation has hindered genetic counseling for the family. Homozygosity of the *UNC80* c.5609-4G>A variant resulted from UPD, thus the recurrence risk for IHPRF2 in this family is significantly reduced compared to a 25% risk expected when both parents are carriers of a *UNC80* pathogenic variant. These findings indicate that UPD is a possible cause of autosomal recessive diseases and should be noticed, especially when patients have large chromosome segments of AOH or a rare homozygous pathogenic variant.

**FIGURE 2:**
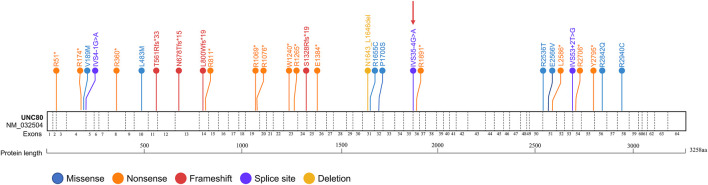
Reported variants in the *UNC80* gene. Structure of *UNC80* gene is shown, along with the 29 previously reported variants. The variant found in the current study is indicated by a red arrow.

Comprehensive clinical and genetic analysis of 39 reported cases of IHPRF2 (including the present case) from 23 families are summarized in Supplementary Table S1. A total of 29 distinct *UNC80* variants were identified from these patients (Supplementary Table S2). These variants included thirteen nonsense, eight missense, four frameshifts, three splice-site and one deletion. Approximately 65.5% (19/29) of the variants were nonsense, frameshift or splicing variants, indicating that loss of function is the mechanism behind the cause of IHPRF2 by *UNC80* variants. Variants were unevenly distributed throughout the *UNC80* gene (Figure 2), but were mainly concentrated in the exons (26/29, 89.66%). Twenty-three variants were observed once or twice, indicating high genetic heterogeneity among IHPRF2 patients. However, approximately 50% of the alleles in IHPRF2 patients were contributed by only 6 variants including p. Arg51*, p. Arg174*, p. Val189Met, c.601-1G>A, p. Thr561Arg fs*33 and p. Arg1265*. Almost all of the 6 variants were identified in the homozygous state in pedigrees with at least 2 patients. Only three genetically diagnosed IHPRF2 patients with compound heterozygous variants were reported from the Chinese population, and they possessed six variants including c.1447C>A, c.3719G>A, c.4926_4937del, c.4963C>T, c.8818C>T, and 8385C>G (Yang et al., 2017; He et al., 2018). These findings did not show a specific hotspot variant, indicating that most IHPRF2 patients in China do not undergo genetic testing.

Genotypes among the 23 genetically diagnosed families were highly heterogeneous, with each family presenting with a unique genotype. Only one variant, c.520C>T (p.Arg174*), was observed in 2 different families. This finding can be attributed to complex phenotypic heterogeneity of IHPRF2. Among the 39 genetically diagnosed patients, 29 (74.36%) presented with homozygous genotypes. Most patients were associated with consanguineous marriages (27/29, 93.10%), which explains why IHPRF2 is highly prevalent in Mediterranean and Arabic populations.

The 39 patients presented with intellectual disability, global developmental delay, absent or very poor speech and dyskinesias. Most patients had facial dysmorphisms including triangular face (31/35), strabismus (24/31), microcephaly (24/35), thin upper lip (16/22), prominent nasal bridge (10/14), short and smooth philtrum (9/13), frontal bossing (9/13), low set posterior rotated ears (9/13) and high forehead (8/9). Other common phenotypes included hypotonia (35/37), tapering fingers (21/24), birth length less than 3rd percentile (20/25), feeding difficulties (17/23), constipation (15/20), and esotropia (11/13). In addition, other neurodevelopmental abnormalities including seizures, abnormal EEG, abnormal brain MRI, and inability to walk independently were observed in 43.24% (16/37), 64.52% (20/31), 43.33% (13/30), and 83.33% (15/18) of IHPRF2 patients, respectively.

In this study, null *UNC80* alleles were defined as those containing frameshift, splice-site, and/or nonsense mutations. Patients were split into two groups with different genotypes including: (i) N/N (null/null) (n = 30) and (ii) M/M (missense/missense) (n = 8). One individual with genotype of M/N (missense/null) was excluded. Incidences of most phenotypes of the individuals with genotype “N/N” werelarger compared to that of the “M/M” group (Table 1), indicating a relatively poor survival in patients with null variants. However, there was no significant difference observed between the two groups which can be attributed to the small sample size, challenges in phenotypic assessment, and other modifying factors that influence IHPRF2 phenotypes. In this study, the patient exhibited a homozygous splice site variant and was grouped in the “N/N” genotype class. He had been unwell since birth, and presented with severe congenital nervous system anomalies as well as global developmental delay. Seizures were not observed, and there was no way to verify that seizures may have actually ever occurred and were not captured. The patient presented with typical clinical manifestations of IHPRF2 and relatively poor prognosis, which can be associated with the null variant.

**TABLE 1:** Clinical features of IHPRF2 patients.

Clinical feature		Frequency in reported affected individuals with pathogenic *UNC80* variants			
		N/N	M/M	Total	This study
Neurodevelopmental and behavioral	Profound intellectual disability	26/26 (100%)	7/7 (100%)	34/34 (100%)	+
	Seizures	11/29 (37.93%)	5/7 (71.43%)	16/37 (43.24%)	−
	Feeding difficulties	16/19 (84.21%)	2/3 (66.67%)	18/23 (78.26%)	+
	Absent or very poor speech	27/27 (100%)	7/7 (100%)	34/34 (100%)	+
	Dyskinesias	23/23 (100%)	4/4 (100%)	28/28 (100%)	+
	hypotonia	27/29 (93.10%)	7/7 (100%)	35/37 (94.59%)	+
	Inability to walk independently	12/13 (92.31%)	3/4 (75%)	16/18 (88.89%)	+
	Abnormal brain MRI	12/25 (48%)	1/4 (25%)	13/30 (43.33%)	+
	Abnormal EEG	15/25 (60%)	5/5 (100%)	20/31 (64.52%)	−
Ophthalmologic	Strabismus	22/27 (81.48%)	1/3 (33.33%)	24/31 (77.42%)	+
	Esotropia	11/12 (91.67%)	1/2 (50%)	11/13 (84.62%)	+
Growth	Normal birth parameters	18/22 (81.82%)	5/6 (83.33%)	24/29 (82.67%)	+
	Height < 3rd Percentile	15/18 (83.33%)	2/6 (33.33%)	18/25 (72%)	+
	Weight < 3rd Percentile	16/19 (84.21%)	3/6 (50%)	20/25 (80%)	−
	Severe global developmental delay	30/30 (100%)	7/7 (100%)	38/38 (100%)	+
	IUGR	9/12 (75%)		9/13 (69.23%)	−
Nonspecific facial features	Triangular face	26/29 (89.66%)	4/5 (80%)	31/35 (88.57%)	+
	Micrognathia	6/12 (50%)		7/13 (53.85%)	−
	Microcephaly	20/28 (71.43%)	3/6 (50%)	24/35 (68.57%)	+
	High forehead	8/9 (88.89%)		8/9 (88.89%)	−
	Frontal bossing	8/12 (66.67%)		9/13 (69.23%)	−
	Short and smooth philtrum	9/12 (75%)		9/13 (69.23%)	−
	Low set posterior rotated ears	9/12 (75%)		9/13 (69.23%)	+
	Nystagmus	1/7 (14.29%)	0/3 (0%)	1/10 (10%)	−
	Prominent nasal bridge	10/13 (76.92%)		10/14 (76.92%)	−
	Thin upper lip	16/20 (80%)	0/2 (0%)	16/22 (72.73%)	+
	Tented upper lip	7/8 (87.50%)		7/8 (87.50%)	+
Musculoskeletal	Tapering fingers	21/23 (91.30%)		21/24 (87.50%)	+
	Clubfeet	6/11 (54.55%)		6/11 (54.55%)	−
	Scoliosis	10/14 (71.43%)	1/3 (33.33%)	11/18 (61.11%)	−
	Contractures	10/24 (41.67%)		10/24 (41.67%)	−
Gastrointestinal	Constipation	13/14 (92.86%)	2/3 (66.67%)	15/20 (75%)	+

M, missense allele; N, null allele, null alleles were defined as those containing frameshift, splice-site and/or nonsense variants.

In summary, a novel splicing variant, c.5609-4G>A in *UNC80* was identified in the current study. This finding elucidatesn on genetic variants that cause IHPRF2. The review section describes all known *UNC80* variants, thus provides a basis for exploring genotype-phenotype correlations of IHPRF2. The findings of this current study indicate the importance of genetic testing in identifying the underlying molecular cause of the IHPRF2 disease and in providing adequate genetic counseling on possible recurrence risks.

## Data Availability

The datasets presented in this study can be found in online repositories. The names of the repository/repositories and accession number(s) can be found below: NCBI SRA BioProject, accession no: PRJNA757139.
